# Serum biomarkers from cell-based assays for AhRL and MIS strongly predicted the future development of diabetes in a large community-based prospective study in Korea

**DOI:** 10.1038/s41598-020-62550-6

**Published:** 2020-04-14

**Authors:** Hong Kyu Lee, Wook Ha Park, Young Cheol Kang, Sora Kang, Suyeol Im, Sol Park, Jin Taek Kim, Minhyeok Lee, Junhee Seok, Man-Suk Oh, Hoon Sung Choi, Youngmi Kim Pak

**Affiliations:** 1 Department of Internal Medicine, Eulji University College of Medicine, Seoul, Korea; 2 Department of Physiology, College of Medicine, Kyung Hee University, Seoul, Korea; 3 Department of Neuroscience, Medical Research Center for Bioreaction to Reactive Oxygen Species and Biomedical Science Institute, School of Medicine, Graduate School, Kyung Hee University, Seoul, Korea; 4 Department of Statistics, College of Natural Science, Ewha Womans University, Seoul, Korea; 5 School of Electrical Engineering, Korea University, Seoul, Korea; 6 Department of Internal Medicine, Kangwon National University School of Medicine, Chuncheon-si, Korea

**Keywords:** Biomarkers, Predictive markers, Endocrine system and metabolic diseases, Risk factors

## Abstract

Exposure to environment-polluting chemicals (EPC) is associated with the development of diabetes. Many EPCs exert toxic effects via aryl hydrocarbon receptor (AhR) and/or mitochondrial inhibition. Here we investigated if the levels of human exposure to a mixture of EPC and/or mitochondrial inhibitors could predict the development of diabetes in a prospective study, the Korean Genome and Epidemiological Study (KoGES). We analysed AhR ligands (AhRL) and mitochondria-inhibiting substances (MIS) in serum samples (n = 1,537), collected during the 2008 Ansung KoGES survey with a 4-year-follow-up. Serum AhRL, determined by the AhR-dependent luciferase reporter assay, represents the contamination level of AhR ligand mixture in serum. Serum levels of MIS, analysed indirectly by MIS-ATP or MIS-ROS, are the serum MIS-induced mitochondria inhibiting effects on ATP content or reactive oxygen species (ROS) production in the cultured cells. Among 919 normal subjects at baseline, 7.1% developed impaired glucose tolerance (IGT) and 1.6% diabetes after 4 years. At the baseline, diabetic and IGT sera displayed higher AhRL and MIS than normal sera, which correlated with indices of insulin resistance. When the subjects were classified according to ROC cut-off values, fully adjusted relative risks of diabetes development within 4 years were 7.60 (95% CI, 4.23–13.64), 4.27 (95% CI, 2.38–7.64), and 21.11 (95% CI, 8.46–52.67) for AhRL ≥ 2.70 pM, MIS-ATP ≤ 88.1%, and both, respectively. Gender analysis revealed that male subjects with AhRL ≥ 2.70 pM or MIS-ATP ≤ 88.1% showed higher risk than female subjects. High serum levels of AhRL and/or MIS strongly predict the future development of diabetes, suggesting that the accumulation of AhR ligands and/or mitochondrial inhibitors in body may play an important role in the pathogenesis of diabetes.

## Introduction

Exposure to multiple environment-polluting chemicals (EPC) is becoming increasingly important to understand the pathogenesis of metabolic disease and diabetes epidemics^1–3^. Many epidemiological studies have shown that high serum concentrations of EPCs are strongly associated with obesity^4^, diabetes^5^, and metabolic syndrome^6^. Many endocrine-disrupting chemicals (EDCs)^7,8^ and metabolism-disrupting chemicals (MDCs)^2^ are listed as causes of obesity and diabetes, and thus referred to as *obesogens* and *diabetogens*. However, establishing the cause-effect relationship between exposure to these chemicals and development of obesity or diabetes in humans have been inconsistent^9^. Among the many reasons behind this, the limitation of the methods estimating the exposure level to EPCs seems to be the most important^10,11^. Humans are exposed to an immense variety of chemicals^10^, with the nature of these mixtures differing between populations and over time. Experimental studies have shown that EPC mixtures have substantial toxic effects *in vivo* at concentrations 100-fold or more below their no-observed-adverse-effect-levels (NOAELs)^11^, but little is known about the health risks of the EPC mixture in humans^10^. Understanding the link between EPC mixtures and the diseases requires large prospective studies with a series of measurements of a wide range of EPCs.

Having successfully determined the levels of human exposure to EPC mixtures by *in vitro* incubation of the cultured aryl hydrocarbon receptor (AhR)-dependent luciferase reporter cells with human serum samples^12,13^, we applied these bioassays to the prospective epidemiologic study. Since many of these EPC, EDC, and MDC chemicals are ligands of AhR transcription factor, serum AhR ligand (AhRL)-mediated luciferase bioactivity can be used as a biomarker for exposure level to EPC mixtures composed of various AhR ligands^14^ in humans. Our previous studies showed that serum AhRL, which was linearly correlated with the toxic equivalency (TEQ) value of the tested persistent organic pollutant (POP) mixtures, was higher in Koreans with impaired glucose tolerance (IGT) and diabetes than those with normal glucose tolerance (NGT)^13^, and was associated with components of metabolic syndrome and insulin resistance^15^. AhRL had a positive correlation with serum insulin and HOMA-IR, and a negative correlation with adiponectin^15^. There is also strong evidence that exposure to EPC can cause mitochondrial dysfunction in cells and animals^16,17^.

Human studies of individuals with insulin resistance, both those with established diabetes and IGT, have consistently demonstrated structural or functional defects in the mitochondria^18,19^. Although it is not clear whether mitochondrial defects are the primary cause or secondary to subtle defects in glucose metabolism, insulin resistance, or impaired insulin secretion in the early stages of disease development^20^, mitochondrial dysfunction is believed to be involved in the pathogenesis of diabetes. Regardless of whether the chemicals in serum were AhR ligands, some of them inhibited mitochondrial activity in cells^12^. Incubating cells with mitochondria-inhibiting substances (MIS) decreases intracellular ATP content and increases DCF-DA-labelled reactive oxygen species (ROS) levels^12^. Thus, the levels of MIS in a serum sample could be indirectly determined by other cell-based assays measuring intracellular ATP concentration and/or ROS production in the cultured cells treated with the human serum sample. The resulting levels of ATP and ROS represent indirectly how much MIS are present in serum samples. To avoid confusion from endogenous ATP and ROS contents in the sample, we defined the outputs of the ATP and ROS assays as MIS-ATP and MIS-ROS, respectively. The resulting MIS-ATP and MIS-ROS levels also significantly correlated with TEQ of the tested POP mixtures^12,13^.

In the current study, we investigated whether serum AhRL, MIS-ATP, and MIS-ROS could predict the future development of IGT and diabetes using serum samples collected from a large, well-characterized, community-based prospective epidemiologic study, the Korean Genome and Epidemiology Study (KoGES)^21^. We report evidence that serum AhRL and MIS are important predictive factors of diabetes. These data suggest the inhibition of mitochondrial function by EPC mixtures might be a key mechanism leading to diabetes.

## Methods

Detailed descriptions of the methods are presented in the Supplementary Methods.

### Study participants

The Ansung cohort of KoGES was established to investigate the genetic and environmental aetiology of common, complex diseases in Koreans. The results of KoGES are open to the public, and a summary of this study was published^22^. The data used in this study were downloaded from the KoGES depository with permission (KoGES; 4851-302). We analysed all the available samples, 1,537 sera collected during oral glucose tolerance test (OGTT) in 2008, to maximize the statistical power. The definitions of NGT, IGT, and diabetes were based on the results of the 75 g OGTT and the WHO criteria^23,24^. We then compared data obtained from samples collected during the 2012 follow-up study.

### AhR ligand (AhRL) bioactivity assay

The pGL4-DRE-luc(puromycin+)/pRL-mTK double-positive stable cells and heat-inactivated serum samples were prepared as described previously^12^. The AhRL assay is similar to the CALUX assay^25^, except utilizing the different recombinant cell lines and organic solvent extraction-free sample preparation method. All cell-based assays were performed in duplicate on blinded samples. The intra- and inter-assay coefficients of variation for AhRL were less than 5.0%.

### MIS-ATP and MIS-ROS assays for serum-induced mitochondria inhibition

Levels of MIS in serum samples were evaluated by measuring intracellular ATP content (MIS-ATP) and ROS generation (MIS-ROS) as described^12,13^. pRL-mTK-transfected mouse Hepa1c1c7 cells (5 × 10^4^/well) in a 96-well plate were treated with 10 μL heat-inactivated-serum samples for 48 h. The ATP content was determined using the CellTiter-Glo luciferase kit (Promega, Madison, WI, USA), with the output being normalized to *Renilla* luciferase activity. ROS level was determined using 5-(and-6)-chloromethyl-2′,7′-dichlorodihydrofluorescein diacetate and acetyl ester (CM-H_2_DCFDA; Molecular Probes, Eugene, OR, USA). Both MIS-ATP and MIS-ROS were expressed as % of charcoal stripped serum (CSS)-treated control. The intra- and inter-assay coefficients of variation for these methods were less than 6.0%.

### Statistical analysis

Results are presented as means ± SD, as numbers and percentages, or as RR with 95% CI. Student’s *t*-tests or ANOVA were used to compare the means of continuous variables among the groups. Tukey *post hoc* tests were performed to assess which pairs of groups showed significant differences. Comparisons of the proportions of categorical variables among the groups were performed using the χ^2^ test or logistic regression. Optimal cut-off values of AhRL and MIS-ATP for the prediction of diabetes development were estimated using receiver-operating characteristics (ROCs) and the Youden method^26^. RRs represented by AhRL or MIS-ATP for diabetes development within 4 years were analysed with logistic regression, with or without adjustment for potentially confounding variables. The measured AhRL, MIS-ATP, and MIS-ROS were clustered using the K-means clustering algorithm^27^.

## Results

### Clinical characteristics and serum biomarkers of the participants

When the participants (n = 1,537) were classified according to glucose tolerance, 919 (59.8%) had NGT, 244 (15.9%) had IGT, and 374 (24.3%) had diabetes. The clinical characteristics of the participants are shown in Supplementary Table S1. The average age of the study subjects was 61.4 ± 8.2 years (men = 45.1%). Most of the parameters related to insulin resistance and inflammation, including BMI, blood pressure, HbA1c, HOMA-β, HOMA-IR, triglycerides, ALT/AST, and hsCRP, of participants in the NGT group were significantly different from those in the IGT or diabetes groups. Smoking status and alcohol intake were slightly higher in diabetes group. Exercise rate was not different among three groups.

**Figure 1:**
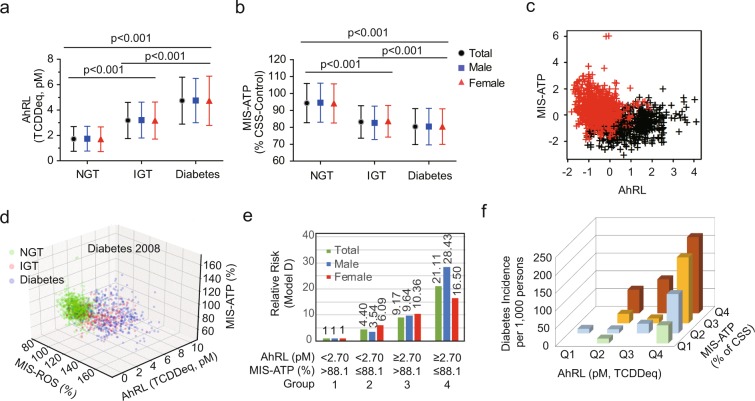
The mean levels of AhRL (TCDDeq, pM) (**a**) and MIS-ATP (% of CSS-treated control) (**b**) according to glucose tolerance state at baseline year 2008. (**c**) Two clusters by K-means clustering in the plot of AhRL *vs*. ATP. (**d**) The 3D scatter plot with AhRL, MIS-ATP and MIS-ROS for 2008 diabetes diagnose. (**e**) Relative risks of diabetes developing within 4 years according to a combination of AhRL and MIS-ATP in the multivariable logistic regression model D (sex, age, smoking, drinking, and exercise, waist circumference, systolic BP, fasting glucose, and triglyceride-adjusted). Non-diabetic subjects (Total = 1,163; male = 503; female = 660) were divided into 4 groups according to their cut-off values of AhRL and MIS-ATP. See Supplementary Table S4 and Fig. S5 for precise values and other models. (**f**) The incidences of new-onset diabetes per 1,000 people, according to the quartiles of AhRL and MIS-ATP. The incidences were calculated for 1,163 non-diabetic subjects at baseline. There were very few non-diabetic subjects in the lowest quartile (Q1) of AhRL. Quartile ranges for AhRL: Q1 ≤ 1.08; 1.08 < Q2 < 1.94; 1.95 < Q3 < 2.61 pM. Quartile ranges for MIS-ATP: Q1 > 98.7%; 98.7% ≥ Q2 > 91.7%; 91.7% ≥ Q3 > 84.2%; Q4 ≤ 84.2%.

Table 1 shows the mean levels of three serum biomarkers for AhRL (TCDDeq, pM), MIS-ATP (% of CSS-treated control), and MIS-ROS (% of CSS-treated control) according to glucose tolerance state at 2008. IGT and diabetes groups showed 1.8–2.8-fold increase of AhRL compared to the NGT group (*p* < 0.001, Supplementary Fig. S1). MIS-ATP of the IGT and diabetes groups were 12–15% lower than the NGT group (*p* < 0.001). MIS-ROS in the IGT and diabetes groups were 10–16% higher than the NGT group (*p* < 0.001). No gender differences were observed in mean levels of AhRL, MIS-ATP, and MIS-ROS (Fig. 1a,b, Supplementary Fig. S1).

**Table 1:** The levels of serum biomarkers of the participants according to glucose tolerance state in year 2008.

	Gender (n)	NGT mean (±SD)	IGT mean (±SD)	Diabetes mean (±SD)	*p* value*	*Post hoc* (Tukey)
N	Total (1,537)	919	244	374		
	Male (687)	399	104	184		
	Female (850)	520	140	190		
AhRL (pM, TCDDeq)	Total	1.72 (0.97)	3.18 (1.43)	4.74 (1.85)	<0.001	a,b,c
	Male	1.74 (0.98)	3.21 (1.41)	4.75 (1.75)	<0.001	a,b,c
	Female	1.70 (0.97)	3.17 (1.46)	4.73 (1.94)	<0.001	a,b,c
MIS-ATP (% Control)	Total	94.3 (11.6)	83.2 (9.6)	80.4 (10.6)	<0.001	a,b,c
	Male	94.6 (11.5)	82.7 (9.9)	80.4 (10.8)	<0.001	a,b
	Female	94.1 (11.6)	83.6 (9.4)	80.4 (10.5)	<0.001	a,b,c
MIS-ROS (% Control)	Total	109.7 (9.3)	122.1 (15.0)	127.3 (19.6)	<0.001	a,b,c
	Male	109.5 (9.4)	122.9 (14.7)	128.5 (19.2)	<0.001	a,b,c
	Female	109.9 (9.3)	121.5 (15.2)	126.0 (19.9)	<0.001	a,b,c

In *post hoc* analysis, each lower-case letter in the last column represents a pair with a significant difference; ‘a’ for NGT and IGT; ‘b’ for NGT and DM; ‘c’ for IGT and DM. AhRL is expressed as 2,3,7,8-tetrachlorodibenzodioxin (TCDD) equivalents (TCDDeq, pM). MIS-ATP and MIS-ROS are expressed as % of the 10% charcoal-stripped human serum (CSS)-treated control. *p values were calculated by one-way analysis of variance, with Tukey’s *post hoc* test used to determine differences among the groups.

### Correlations of AhRL, MIS-ATP and MIS-ROS with clinical parameters in the baseline survey

AhRL across the whole group showed significant positive correlations with age, obesity, systolic blood pressure, blood glucose concentrations, insulin resistance, triglycerides, liver dysfunction, and inflammation, while it showed negative correlations with HOMA β-cell function and HDL (Table 2). Correlation of AhRL with blood glucose levels was moderately strong (r = 0.41~0.51, p < 0.001). MIS-ATP significantly, but negatively correlated with these clinical parameters (r = −0.28 ~ −0.36, p < 0.001). MIS-ROS was significantly and weakly correlated with most of the parameters analysed, but not with age, total cholesterol, or liver dysfunction. These results suggested that AhRL, MIS-ATP, and MIS-ROS may influence the status of obesity, hyperglycemia, insulin resistance, β-cell function, and inflammation of the subjects. It was noted that LDL was correlated with MIS-ATP and MIS-ROS, but not with AhRL. Differences with respect to gender were not observed for the correlations of most parameters, however, only in women, AhRL and MIS-ATP correlated with liver dysfunction (Supplementary Table S2**)**. Since ALT and AST were higher in men than in women, other factors such as smoking or alcohol may affect the increases of ALT and AST in men. AhRL, but not MIS-ATP, was significantly higher in ex-smokers and former alcohol drinkers (Supplementary Table S3). MIS-ROS was significantly higher in ex-smokers and in people that exercised regularly. All other clinical parameters showed weak or moderate correlation with the three biomarkers, implying that they are independent parameters.

**Table 2:** Correlations of AhRL, MIS-ATP, and MIS-ROS with clinical parameters across the whole group.

	AhRL		MIS-ATP		MIS-ROS	
	r	*p* value	r	*p* value	r	*p* value
Age	0.17	<0.001	−0.13	<0.001	0.04	0.139
BMI (kg/m^2^)	0.15	<0.001	−0.10	<0.001	0.10	<0.001
Weight (kg)	0.09	<0.001	−0.06	0.022	0.09	<0.001
Waist circumference (cm)	0.20	<0.001	−0.15	<0.001	0.15	<0.001
Systolic BP (mm Hg)	0.15	<0.001	−0.14	<0.001	0.11	<0.001
Diastolic BP (mm Hg)	0.05	0.048	−0.06	0.027	<0.01	0.970
HbA_1c_ (%)*	0.51	<0.001	−0.29	<0.001	0.23	<0.001
FPG (mmol/L)*	0.41	<0.001	−0.28	<0.001	0.31	<0.001
2 h glucose (mmol/L)*	0.51	<0.001	−0.36	<0.001	0.35	<0.001
Fasting insulin (mmol/L)*	0.15	<0.001	−0.11	<0.001	0.16	<0.001
HOMA-IR*	0.28	<0.001	−0.20	<0.001	0.26	<0.001
HOMA-β*	–0.23	<0.001	0.15	<0.001	−0.13	<0.001
Total cholesterol (mg/dL)	0.03	0.245	0.01	0.627	−0.03	0.188
Triglyceride (mg/dL)*	0.23	<0.001	−0.18	<0.001	0.13	<0.001
HDL cholesterol (mg/dL)*	–0.13	<0.001	0.09	<0.001	−0.12	<0.001
LDL cholesterol (mg/dL)	–0.05	0.071	0.07	0.003	−0.07	0.003
ALT (IU/L)*	0.19	<0.001	−0.12	<0.001	0.03	0.311
AST (IU/L)*	0.12	<0.001	−0.10	<0.001	−0.02	0.443
hsCRP (mg/L)*	0.13	<0.001	−0.06	0.013	0.11	<0.001
AhRL			−0.34	<0.001	0.36	<0.001
MIS-ATP					−0.27	<0.001

Correlation coefficients (r) and p values were calculated using Pearson’s correlation analysis. *Variable was log2-transformed before statistical analysis. BMI, body mass index; FPG, fasting plasma glucose; BP, blood pressure; HOMA-β, homeostasis model assessment of β-cell function; HOMA IR, HOMA insulin resistance; HDL, high-density lipoprotein; LDL, low-density lipoprotein; ALT, alanine aminotransferase; AST, aspartate aminotransferase; hsCRP, high-sensitivity C-reactive protein.

### Correlations between AhRL, MIS-ATP, and MIS-ROS

AhRL correlated with MIS-ATP (r = −0.34, *p* < 0.001) and MIS-ROS (r = 0.36, *p* < 0.001), and MIS-ATP correlated with MIS-ROS (r = −0.27, *p* < 0.001) (Table 2, Supplementary Fig. S2). However, these relationships did not appear to be linear; when AhRL was plotted against MIS-ATP, many subjects had both low MIS-ATP and low AhRL. These findings raise the possibility that there are at least two groups of MIS in the serum: those acting through AhR (AhR ligands) and others acting through non-AhR-mediated mechanisms (non-AhR ligands)^12^. Using K-means clustering analysis, two clusters were identified: a cluster with high AhRB and MIS-ROS, but low MIS-ATP (black dots in Fig. 1c, Supplementary Fig. S2) and another cluster in which AhRB and MIS-ROS decreased alongside MIS-ATP (red dots in in Fig. 1c). The proportion of diabetes within the black cluster in 2008 was significantly higher than in the red cluster (67.2% vs. 8.2%, *p* < 0.001). In 2012, the black cluster had also significantly more diabetes patients than the red cluster (74.6% vs. 11.7%, *p* < 0.001).

The distribution changes of AhRL, MIS-ATP, and MIS-ROS in NGT, IGT, and diabetes groups are illustrated by 3D scatter plots for 2008 (Fig. 1d) and 2012 diabetes diagnoses (Supplementary Fig. S2d). Many of those with high AhRL and MIS-ROS with low MIS-ATP (possibly belong to black cluster) in the NGT or IGT group in 2008 progressed to diabetes in 2012.

### Cut-off values for AhRL, MIS-ATP, and MIS-ROS determining diabetes development

In logistic regression analyses for diabetes development among the 1,163 subjects that did not have diabetes at baseline (NGT [919] + IGT [244]), AhRL, MIS-ATP, and MIS-ROS had significant odds ratios of 2.28 (1.91–2.72), 1.09 (1.05–1.11), and 1.04 (1.02–1.05), respectively. In ROC analyses, the optimal cut-off value of AhRL was 2.70 pM of TCDDeq (AUC = 0.821, sensitivity = 72.5%, specificity = 79.8%) (Supplementary Fig. S3). The optimal cut-off levels of MIS-ATP and MIS-ROS were 88.1% (AUC = 0.742, sensitivity = 73.9%, specificity = 64.4%) and 120% (AUC = 0.600, sensitivity = 44.9%, specificity = 78.7%), respectively. These data indicate that AhRL had good prediction performance and MIS-ATP had fair performance; however, MIS-ROS showed poor performance. Gender-dependent analysis revealed that AhRL was better in predicting diabetes development in men than in women.

### The influence of AhRL and MIS-ATP on the development of IGT or diabetes

Since MIS-ATP is a better indicator than MIS-ROS, MIS-ATP was used as a parameter for mitochondria inhibiting activity in the following analyses. Low MIS-ATP means high levels of MIS exist in serum. The mean levels of AhRL and MIS-ATP are shown in Table 3, with participants classified according to whether they developed IGT or diabetes during the 4-year follow-up period (2008–2012). Of the 919 NGT subjects in 2008, 109 cases (7.1%) and 25 cases (1.6%) in 2012 were progressed to IGT (NGT-IGT) and diabetes (NGT-DM), respectively. These newly-diagnosed IGT (NGT-IGT) and diabetes (NGT-DM) patients showed 60%~70% higher AhRL and 6%~12% lower MIS-ATP than NGT-NGT (*p* < 0.001).

**Table 3:** Mean levels of AhRL and MIS-ATP, classified according to the development of glucose intolerance or diabetes between 2008 and 2012. Gender differences were compared.

(2008–2012)		Cases (% total)	AhRL (pM, TCDDeq)	*p* value	MIS-ATP (% of CSS)	*p* value
NGT, remained with NGT (NGT-NGT)	Total	785 (51.1%)	1.58 ± 0.88	Ref.	95.3 ± 11.5	Ref.
	Male	348 (50.7%)	1.64 ± 0.90	Ref.	95.5 ± 11.2	Ref.
	Female	437 (51.4%)	1.53 ± 0.86	Ref.	95.1 ± 11.7	Ref.
NGT, progressed to IGT (NGT-IGT)	Total	109 (7.1%)	2.52 ± 1.08	<0.001	89.7 ± 9.9	<0.001
	Male	38 (5.5%)	2.45 ± 1.22	<0.001	89.4 ± 11.2	0.002
	Female	71 (8.3%)	2.52 ± 1.00	<0.001	89.9 ± 9.3	<0.001
NGT, progressed to diabetes (NGT-DM)	Total	25 (1.6%)	2.69 ± 0.98	<0.001	83.5 ± 10.7	<0.001
	Male	13 (1.9%)	2.71 ± 0.90	<0.001	84.8 ± 13.3	0.013
	Female	12 (1.4%)	2.67 ± 1.10	<0.001	82.7 ± 7.4	<0.001
IGT reverted to NGT (IGT-NGT)	Total	125 (8.1%)	2.64 ± 1.31	<0.001 [ref]	84.3 ± 10.4	<0.001 [ref]
	Male	56 (8.1%)	2.66 ± 1.32	<0.001 [ref]	84.7 ± 11.0	<0.001 [ref]
	Female	69 (8.1%)	2.62 ± 1.31	<0.001 [ref]	84.9 ± 9.9	<0.001 [ref]
IGT, remained in IGT (IGT-IGT)	Total	75 (4.9%)	3.59 ± 1.27	<0.001 [<0.001]	81.9 ± 8.3	<0.001 [0.070]
	Male	21 (3.1%)	3.51 ± 1.08	<0.001	79.1 ± 7.22	<0.001
	Female	54 (6.4%)	3.62 ± 1.35	<0.001	82.9 ± 8.5	<0.001
IGT, progressed to diabetes (IGT-DM)	Total	44 (2.9%)	4.04 ± 1.41	<0.001 [<0.001]	82.3 ± 9.1	<0.001 [<0.265]
	Male	27 (3.9%)	4.11 ± 1.30	<0.001 [<0.002]	81.2 ± 8.3	<0.001 [0.111]
	Female	17 (2.0%)	3.92 ± 1.61	<0.001	84.1 ± 10.3	<0.001 [0.956]
Remained in diabetes (DM)	Total	374 (24.3%)	4.74 ± 1.85	<0.001	80.4 ± 10.6	<0.001
	Male	184 (26.8%)	4.75 ± 1.75	<0.001	80.4 ± 10.8	<0.001
	Female	190 (22.4%)	4.73 ± 1.94	<0.001	80.4 ± 10.5	<0.001

AhRL is expressed as 2,3,7,8-tetrachlorodibenzodioxin (TCDD) equivalents (TCDDeq, pM). MIS-ATP is expressed as a percentage of the 10% charcoal-stripped human serum (CSS)-treated control. Values are expressed as mean ± standard deviation. *p* values were calculated using Students t-test between subjects with NGT that remained with NGT and the other groups. *p* values in brackets represent comparisons with the IGT, reverted to NGT group.

Among the 244 IGT subjects in 2008, 125 cases (8.1%) reverted to NGT (IGT-NGT), 44 cases (2.9%) developed diabetes (IGT-DM), and 75 cases (4.9%) maintained IGT (IGT-IGT) in 2012. Similar to results from NGT group, AhRL of diabetes (IGT-DM) and IGT (IGT-IGT) were 36%~53% higher than NGT reverters (IGT-NGT) (*p* < 0.001). The difference of MIS-ATP was only 2~3% between IGT (IGT-IGT) or diabetes (IGT-DM) and NGT reverters (IGT-NGT) (*p* < 0.001). Supplementary Fig. S4 is a graphical summary of the AhRL and MIS-ATP for NGT, IGT, and diabetes groups in 2012 among NGT, IGT and diabetes groups in 2008. In all groups, AhRL was NGT < IGT < diabetes, but MIS-ATP was NGT > IGT > diabetes. There was no gender-difference in levels of AhRL or MIS-ATP among IGT or diabetes progressors. Compared with NGT-NGT group, subjects of IGT in 2008 already had significantly higher levels of AhRL and lower levels of MIS-ATP regardless of the disease progression. This suggests that AhR ligands and unidentified MIS may be interacting in the progression of IGT and diabetes.

### Effects of AhRL and MIS-ATP on the incidence and risk of new-onset diabetes

In a multivariate logistic regression model, the relative risk (RR) for new-onset diabetes during the 4-year follow-up period was calculated among non-diabetic subjects (n = 1,163). For subjects with an AhRL cutoff value ≥ 2.70 pM, the RR was 10.40 (95% CI, 6.01‒17.99) in the unadjusted model (Model A), compared with those with an AhRL < 2.70 pM. The increased risk for subjects with AhRL ≥ 2.70 pM remained highly statistically significant (RR = 7.60, 95% CI, 4.23–13.64) after additional adjustment for all confounding factors (Model D) (Table 4). Similarly, the subjects with MIS-ATP ≤ 88.1% had a high risk of diabetes. RR was 5.09 (95% CI, 2.94–8.84) and 4.27 (95% CI, 2.38–7.64) in Model A and Model D, respectively. Interestingly, men with AhRL ≥ 2.70 pM or MIS-ATP ≤ 88.1% showed higher diabetic risk than women.

**Table 4:** Relative risk (95% CI) of diabetes developing within 4 years of non-diabetic subjects (n = 1,163) over the cutoff values of AhRL or MIS-ATP.

	Total		Male		Female	
**AhRL (pM)**	<2.70	≥2.70	<2.70	≥2.70	<2.70	≥2.70
Total cases	892	271	376	127	516	144
Newly developed diabetes (%)	19 (2.1%)	50 (18.5%)	9 (2.4%)	31 (24.4%)	10 (1.9%)	19 (13.2%)
Model A	1	10.40 (6.01‒17.99)	1	13.17 (6.06‒28.59)	1	7.69 (3.49‒16.95)
Model B	1	10.57 (6.05‒18.48)	1	14.11 (6.44‒30.95)	1	7.11 (3.14‒16.08)
Model C	1	9.40 (5.32‒16.61)	1	14.15 (6.27‒31.90)	1	6.02 (2.62‒13.84)
Model D	1	7.60 (4.23‒13.64)	1	12.66 (5.42‒29.60)	1	4.45 (1.89‒10.47)
**MIS-ATP (%)**	> 88.1	≤88.1	>88.1	≤88.1	>88.1	≤88.1
Total cases	721	442	313	190	408	252
Newly developed diabetes (%)	18 (2.5%)	51 (11.5%)	10 (3.2%)	30 (15.8%)	8 (2.0%)	21 (8.3%)
Model A	1	5.09 (2.94–8.84)	1	5.68 (2.70–11.92)	1	4.55 (1.98–10.43)
Model B	1	4.90 (2.81–8.55)	1	5.54 (2.63–11.68)	1	4.11 (1.76–9.59)
Model C	1	4.71 (2.68–8.29)	1	5.61 (2.62–12.00)	1	3.91 (1.66–9.22)
Model D	1	4.27 (2.38–7.64)	1	6.07 (2.70–13.48)	1	3.03 (1.25–7.35)

Model A: unadjusted. Model B: sex, age, smoking. Model C: model B + waist circumference and systolic BP-adjusted. Model D: model C + fasting glucose and triglyceride-adjusted. AhRL is expressed as 2,3,7,8-tetrachlorodibenzodioxin (TCDD) equivalents (TCDDeq, pM). MIS-ATP is expressed as % of the 10% charcoal-stripped human serum (CSS)-treated control.

Non-diabetic subjects (n = 1,163) were then divided into four groups according to the cut-off values of AhRL and MIS-ATP to determine how the AhRL and MIS-ATP interact in the future development of diabetes: group 1 with AhRL < 2.70 pM and MIS-ATP > 88.1%, group 2 with AhRL < 2.70 pM and MIS-ATP ≤ 88.1%, group 3 with AhRL ≥ 2.70 pM and MIS-ATP > 88.1%, and group 4 with AhRL ≥ 2.70 pM and MIS-ATP ≤ 88.1% (Supplementary Table S4, Supplementary Fig. S5). Compared with group 1, the RRs of developing diabetes in group 4 was 29.82 (95% CI, 12.35–71.99), and 21.11 (95% CI, 8.46–52.67), respectively, in Model A and Model D (Table 5). The subjects in group 3 (high AhRL and high MIS-ATP) were more at risk (RR = 13.45, 95% CI, 4.93–36.72 in Model A) than those in group 2 (low AhRL and low MIS-ATP) (RR = 5.08, 95% CI, 1.91–13.51 in model A). Thus, although MIS-ATP is also an independently important parameter, high-level exposure to AhR ligands may be more important than mitochondrial inhibition in diabetes incidence. Again, men showed higher RR than women in group 4 (model D), suggesting that gender-dependent effects on confounding factors may be present (Fig. 1e, Table 5).

**Table 5:** Relative risk (95% CI) of diabetes developing within 4 years on the combined effect of AhRL ≥ 2.70 pM and MIS ATP ≤ 88.1% (Group 4).

	Total	Male	Female
Total number	168	84	84
Number of diabetes (%)	38 (22.6%)	25 (29.8%)	13 (15.5%)
Model A	29.82 (12.35–71.99)	28.18 (9.45–84.02)	31.68 (6.99–143.45)
Model B	29.34 (12.05–71.44)	29.36 (9.76–88.35)	28.33 (6.17–129.95)
Model C	26.12 (10.63–64.17)	30.35 (9.80–94.02)	23.99 (5.13–112.24)
Model D	21.11 (8.46–52.67)	28.43 (8.83–91.58)	16.50 (3.42–79.67)

Model A: unadjusted. Model B: sex, age, smoking. Model C: model B + waist circumference and systolic BP-adjusted. Model D: model C + fasting glucose and triglyceride-adjusted. AhRL is expressed as 2,3,7,8-tetrachlorodibenzodioxin (TCDD) equivalents (TCDDeq, pM). MIS-ATP is expressed as % of the 10% charcoal-stripped human serum (CSS)-treated control.

When the incidence of new-onset diabetes was analysed according to the quartiles of AhRL or MIS-ATP among the 1,163 non-diabetics, most subjects in the upper fourth quartile (Q4) of AhRL (≥2.62 pM) developed diabetes 4 years later (Fig. 1f). In contrast, few subjects in the lowest quartile (Q1) of AhRL (<1.08 pM) developed diabetes. The incidence of diabetes in the Q2 and Q3 quartiles of AhRL increased as the MIS-ATP decreased. As in the upper fourth quartile (Q4) of AhRL, almost all subjects in the lower fourth quartile (Q4) of MIS-ATP (≤84.1%) developed diabetes regardless of their AhRL levels. Again, these results indicate that AhRL and MIS-ATP are strong, independent but interacting, and predictive factors for diabetes development.

## Discussion

In this study, human exposure to EPC mixture of AhR ligands (AhRL) and MIS (MIS-ATP or MIS-ROS) was measured using serum samples obtained from KoGES study to see if it could be used as a predictor of the incidence of diabetes. Among NGT participants, those who developed into diabetes (NGT-DM) or IGT (NGT-IGT) in 2012 had higher AhRL and lower MIS-ATP in serum than those who maintained NGT (NGT-NGT). People with diabetes were characterized by the highest AhRL and the lowest MIS-ATP in serum (Table 1). AhRL and MIS-ATP were significantly correlated with insulin resistance index and the metabolic syndrome components (Supplementary Table S2). For those with AhRL ≥ 2.70 pM (cutoff value), the RR of diabetes developed within 4 years was 7.60, and for those with MIS-ATP ≤ 88.1%, the RR was 4.27 after adjustment of confounding factors (Model D, Table 4). These results are in good agreement with the hypothesis that exposure to AhR ligands and/or mitochondrial inhibiting substances are important risk factors for insulin resistance, metabolic syndrome, and diabetes.

The ligand-activated transcription factor AhR is multifunctional nuclear receptor, playing a pivotal role in mediating the toxic responses induced by POPs and in detoxifying xenobiotics^28^. The toxicities of POPs have been estimated relative to 2,3,7,8-tetrachlorodibenzodioxin (TCDD; toxic equivalency factor = 1) and total POP-induced toxicity is expressed as TEQ, the sum of each chemical’s concentration multiplied by its toxic equivalency factor value, which reflects the ability to activate AhR^29,30^. Thus, serum concentrations of individual POPs should be measured by instrumental analysis to calculate TEQ. Since there are many known and unknown AhR ligands^31^, it is difficult to measure all AhR ligands present in serum samples. Previously, we had shown that the serum AhRL correlated linearly with the TEQ values calculated from the plasma concentrations of the plasma concentrations of dioxin congeners, suggesting that serum AhRL represents an entire circulating AhR ligand mixture^12,13^. Thus, our AhRL assay can be used as an alternative to TEQ assessment without measuring EPC individually, although serum AhRL was not exactly equivalent to serum TEQ values.

Cells cultured with diabetic serum containing high levels of AhR ligands caused mitochondrial dysfunction, i.e. inhibited mitochondrial oxygen consumption rate and intracellular ATP, and increased ROS and mitochondrial fragmentation^13^. Among polychlorinated biphenyls in serum, both AhR ligands and non-AhR ligands appeared to have contributed to the reduction in mitochondrial activity^12^. Non-AhR ligands, for example, tetrabromobisphenol A^32^ and perfluorooctanoic acid^33^, induce mitochondrial dysfunction, ROS production, and inflammatory responses in pancreatic β-cells or osteoblasts. Indeed, there is a complex relationship among serum AhRL, MIS-ATP, and MIS-ROS. The correlation coefficients between AhRL with MIS-ATP or MIS-ROS were not very high (r = −0.34 for MIS-ATP; r = 0.36 for MIS-ROS, *p* < 0.001) (Table 2). It suggests they are possibly independent contributors or variables. The relationships between AhRL, MIS-ATP, and MIS-ROS were non-linear, and the data-points appeared as two clusters (Fig. 1c, Supplementary Fig. S2). These results suggest the possibility of two groups of mitochondrial inhibitors, either AhR-dependent or AhR-independent, exist in serum samples. AhR ligands with mitochondrial inhibitory activity may be diabetogen or obesogen. Given the large number of AhR ligands and their biological effects^14^, further experimental studies are needed to account for these relationships.

Next, it should be noted that the combination of AhRL and MIS-ATP parameters strongly predicted the future development of diabetes (Fig. 1e, Supplementary Table S4). In subjects with AhRL≥2.70 pM and MIS-ATP ≤ 88.1% (group 4), the incidence of diabetes was 22.6% (38 out of 168), and RRs were 29.82 and 21.11 in Model A and in Model D, respectively (Table 5). In comparison, from a cross-sectional study in the U.S (the National Health and Nutrition Examination Survey), Lee *et al*. reported that among the participants with the highest exposure to 6 persistent organic pollutants (POPs; Σ6POPs), the risk of diabetes was 38 times higher than that among the least exposed group^34^. When four POPs were analysed in the Helsinki Birth Cohort Study, Airaksien *et al*. reported that the risk of developing diabetes in the highest exposed group was 1.64~2.24 times higher than in the least exposed group^35^. The ORs for diabetic risk genes were less than 1.7 in genome-wide association studies^36^. A 2-fold higher ceramide-24 level was associated with 1.45 times higher risk of incident impaired fasting glucose^37^. We believe that an important factor in explaining why RRs in this study were so high was the measurement of AhRL and MIS with the cell-based bioassays.

According to another study of the KoGES cohort, a progressive decline in β-cell function and insulin sensitivity is a crucial factor in the deterioration of glucose tolerance and the development of diabetes^21^. Table 2 shows that HOMA-β-cell function correlated negatively with AhRL and positively with MIS-ATP, whereas HOMA-IR correlated positively with AhRL and negatively with MIS-ATP. This suggests that high AhRL and/or low MIS-ATP may be involved in the impairment of insulin secretion and insulin resistance. This agrees well with our previous observation that AhRL was negatively associated with serum adiponectin concentration^15^. Indeed, AhRL and MIS-ATP were also correlated with hsCRP and with serum ALT and AST. Both adiponectin and hsCRP are well-known risk factors for insulin resistance^38^. These correlations mean that AhR ligands and/or MIS may play an important role in the development of chronic inflammation and fatty liver disease, both of which are common in metabolic syndrome. Other experimental evidence also supports the hypothesis that dioxins impair β-cell function^39,40^ or induces vascular inflammation^41^ in mice. Proteomic analysis of mitochondria from skeletal muscles of diabetes patients showed that subunits of oxidative phosphorylation were downregulated^42^. Transcriptome profiling revealed that a number of genes responsible for mitochondria ATP synthesis, oxidative phosphorylation, or mitochondrial dynamics were downregulated in human diabetes islets or β-cells^43,44^. Inflammatory cytokines were elevated before the onset of diabetes^45^. However, triggering mechanisms of mitochondrial dysfunction and/or inflammation in diabetes are still ill-understood. Chronic hyperglycaemia^46^ was recently suggested to start a vicious cycle of progressive deterioration of β-cells by impairing insulin secretion and mitochondria. However, our study clearly demonstrated that non-diabetic subjects developed diabetes within 4 years when they showed high levels AhRL and/or MIS in serum (Fig. 1e, Table 4). Therefore, if “exposure to high EPCs” is inserted instead of “chronic hyperglycaemia”, many unanswered questions about the pathogenic mechanism of diabetes can be explained, namely, EPCs (AhR ligands or MIS) impair mitochondria in β-cell, skeletal muscle, or liver, resulting in insulin resistance and metabolic dysregulation.

Finally, our data showed that AhRL and mitochondrial dysfunction increased with age. Lipophilic EPC tends to remain in the human body and bioaccumulate when entering through food, air, or other routes. Lifelong accumulation of EPCs in the human body could account for the high prevalence of metabolic syndrome and diabetes in the elderly. A comprehensive worldwide literature review has shown that age is positively associated with most dioxin congeners and their TEQs in the unexposed general adult population^47^. The increased levels of AhR ligands and MIS in circulation had temporal associations with clinical phenotypes, including behavioural factors such as smoking and lack of exercise.

In summary, we show people who have high serum AhRL and/or low MIS-ATP are at high risk of developing diabetes, and AhRL and MIS-ATP have more predictive power when combined. Serum AhRL and/or MIS are tightly associated with insulin resistance, metabolic syndrome, and diabetes and may play an important role in their pathogenesis. Further studies are needed to confirm essential findings of this study in other cohorts or in experimental models.

## Supplementary information

Supplementary information.
