# Supplementary material for: Serum biomarkers from cell-based assays for AhRL and MIS strongly predicted the future development of diabetes in a large community-based prospective study in Korea
Source: Sci Rep. 2020 Apr 14;10:6339. doi: 10.1038/s41598-020-62550-6 (PMC7156500; doi:10.1038/s41598-020-62550-6)
Supplement: Supplementary file 1 — Supplementary information. [file 41598_2020_62550_MOESM1_ESM.docx]

**Supplementary Information for**

**Serum biomarkers from cell-based assays for AhRL and MIS strongly predicted the future development of diabetes in a large community-based prospective study in Korea**

Hong Kyu Lee et al.

**Supplementary Figures**


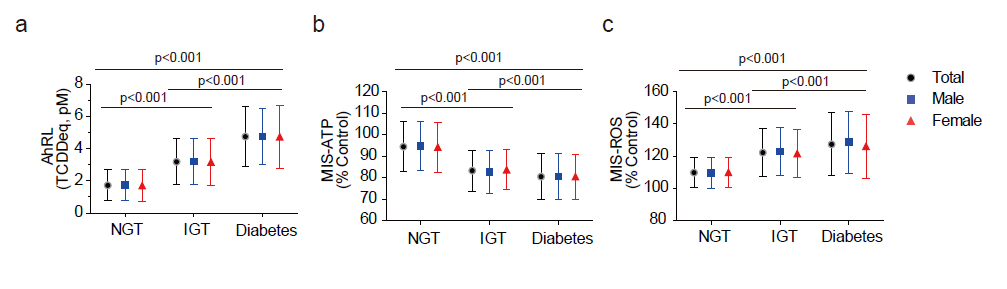


**Supplementary Fig. S1.** Graphical illustration of Table 1. The mean levels of three serum biomarkers according to glucose tolerance state at baseline year 2008. (a) AhRL (TCDDeq, pM), (b) MIS-ATP (% of CSS-treated control), (c) MIS-ROS (% of CSS-treated control). *P* values were calculated by one-way analysis of variance, with Tukey’s *post hoc* test used to determine differences among the groups. No gender differences were observed.


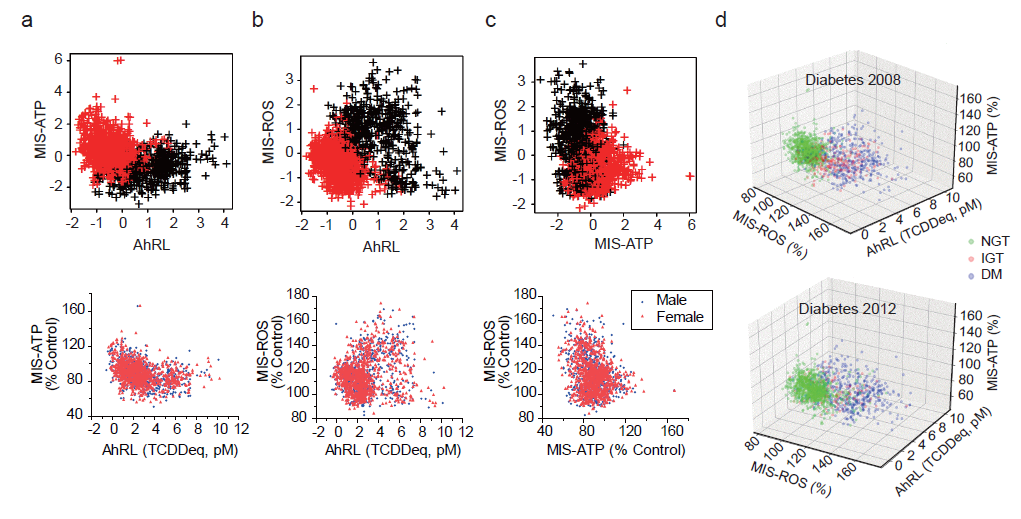


**Supplementary Figure S2. Identification of two clusters of subjects using K-means clustering**

Upper, K-means clustering; Lower, Pearson correlations. (a) AhRL *vs*. MIS-ATP (b) AhRL *vs*. MIS-ROS (c) MIS-ATP *vs*. MIS-ROS. Subjects in the red cluster tended to belong to the NGT group, while those in the black cluster tended to belong to the IGT or diabetes group. (d) The 3D scatter plots with AhRL, MIS-ATP and MIS-ROS in groups of NGT, IGT, and diabetes for 2008 and 2012 diabetes diagnoses.


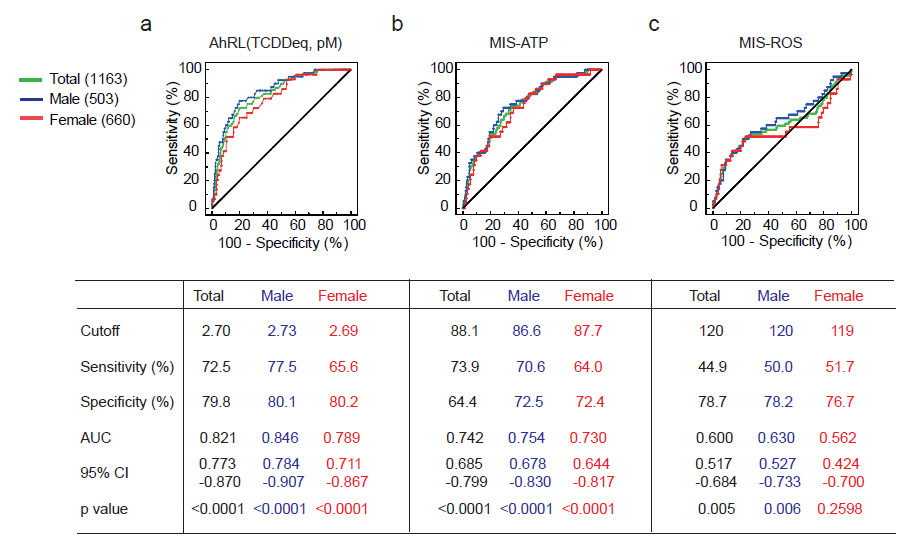


**Supplementary Fig. S3. ROC analysis of AhRL, MIS-ATP, and MIS-ROS for the prediction of diabetes development among subjects (n=1,163) who did not have diabetes at baseline**

(a) AhRL (pM, TCDDeq) (b) MIS-ATP (c) MIS-ROS. Values of AUC, cutoff, sensitivity, and specificity are presented in Table. ROC analysis was performed among total subjects (n=1,163), male (n=503) or female (n=660), who did not have diabetes at baseline. AUC, area under the curve (95% confidence interval).


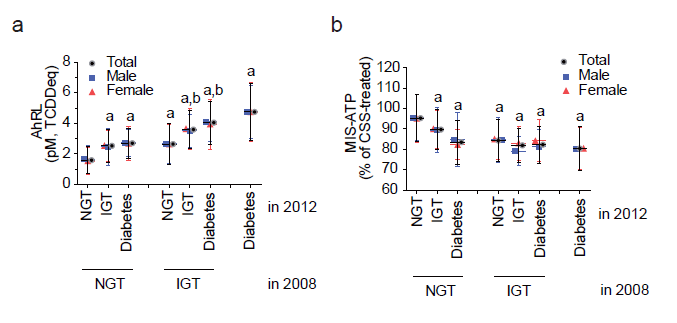


**Supplementary Fig. S4. The mean levels of AhRL and MIS-ATP, classified according to whether subjects developed glucose intolerance or diabetes between 2008 and 2012**

(a) Serum AhRL (TCDDeq, pM) and (b) MIS-ATP levels were compared among the NGT, IGT, and diabetes groups in 2008. Each group was divided into three subgroups according to their status in 2012. Gender-dependent analysis was also performed among total subjects (n=1,537), male (n=687) or female (n=850) (see Table 3 in detail). AhRL is expressed as 2,3,7,8-tetrachlorodibenzodioxin (TCDD) equivalents (TCDDeq, pM). MIS-ATP is expressed as % of the CSS-treated control. Values are expressed as mean ± SEM. The letters ‘a’ and ‘b’ mean that the group is statistically different from NGT-NGT group and the IGT-NGT group, respectively (*p*<0.001).


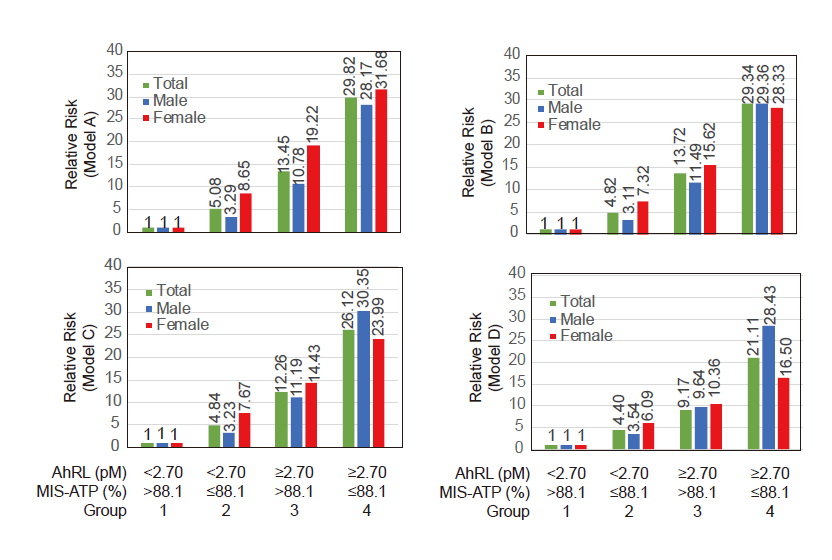


**Supplementary Fig. S5. Combined effects of AhRL and MIS-ATP on relative risks (95% CI) of diabetes incidence within 4 years.** Relative risks of diabetes developing within 4 years according to a combination of AhRL and MIS-ATP in the multivariable logistic regression model. Non-diabetic subjects (Total=1,163; male=503; female=660) were divided into 4 groups according to their cutoff values of AhRL and MIS-ATP. See Supplementary Table S4 for precise values. Model A: unadjusted. Model B: sex, age, smoking, drinking, and exercise adjusted. Model C: model B + waist circumference and systolic BP adjusted. Model D: model C + fasting glucose and triglyceride –adjusted

**Supplementary Table S1. Clinical characteristics of the participants (n=1,537) according to glucose tolerance state at baseline year 2008**

|  | NGT (n=919)  mean (±SD) | IGT (n=244)  mean (±SD) | Diabetes (n=374)  mean (±SD) | *p* value* | *Post hoc*  (Tukey) |
| --- | --- | --- | --- | --- | --- |
| Age (years) | 59.19 (8.34) | 61.84 (8.17) | 63.28 (8.17) | <0.001 | a,b |
| Sex (male %) | 399 (43.42) | 104 (42.62) | 184 (49.20) | 0.129 |  |
| Height (cm) | 159.37 (8.92) | 157.93 (8.97) | 158.63 (9.00) | 0.059 |  |
| Weight (kg) | 60.80 (9.99) | 62.58 (9.91) | 62.90 (10.15) | 0.001 | a,b |
| BMI (kg/m^2^) | 23.89 (3.13) | 25.03 (2.94) | 24.97 (3.34) | <0.001 | a,b |
| Waist circumference (cm) | 86.80 (8.48) | 90.46 (7.91) | 91.39 (8.51) | <0.001 | a,b |
| Hip circumference (cm) | 92.24 (5.38) | 93.29 (4.94) | 92.92 (5.34) | 0.008 | a |
| Systolic BP (mm Hg) | 118.77 (15.49) | 123.48 (16.10) | 126.04 (16.11) | <0.001 | a,b |
| Diastolic BP (mm Hg) | 75.33 (8.79) | 77.35 (9.14) | 76.34 (8.53) | 0.003 | a |
| FPG (mmol/L)^a^ | 5.13 (0.47) | 5.48 (0.53) | 7.28 (2.81) | <0.001 | a,b,c |
| 2h glucose (mmol/L)^a^ | 5.77 (1.16) | 8.89 (0.92) | 11.53 (3.69) | <0.001 | a,b,c |
| $\mathbf{HbA}_{\mathbf{1c}}$ (%)^a^ | 5.45 (0.37) | 5.69 (0.41) | 6.90 (1.36) | <0.001 | a,b,c |
| Fasting insulin (µIU/mL)^a^ | 8.80 (5.20) | 10.01 (5.28) | 11.67 (9.06) | <0.001 | a,b,c |
| IGI_60_ | 0.67 (2.31) | 0.38 (0.67) | 0.46 (4.38) | 0.214 |  |
| HOMA-β^a^ | 113.98 (69.07) | 106.55 (58.05) | 88.32 (110.24) | <0.001 | b,c |
| Composite ISI^a^ | 11.51 (7.25) | 9.34 (5.64) | 8.19 (5.15) | <0.001 | a,b,c |
| HOMA-IR^a^ | 2.02 (1.26) | 2.46 (1.38) | 3.86 (3.79) | <0.001 | a,b,c |
| Total cholesterol (mg/dL) | 190.85 (32.09) | 195.03 (35.57) | 188.20 (34.85) | 0.045 | c |
| HDL-cholesterol (mg/dL)^a^ | 46.71 (10.93) | 43.69 (9.25) | 43.23 (9.54) | <0.001 | a,b |
| LDL-cholesterol (mg/dL) | 119.61 (28.85) | 117.73 (33.66) | 112.11 (32.09) | <0.001 | b |
| Triglycerides (mg/dL)^a^ | 122.63 (65.09) | 168.07 (114.14) | 164.27 (96.34) | <0.001 | a,b |
| Hypertension (%) | 92 (10.0%) | 44 (18.0%) | 75 (20.1%) | <0.001 | a,b |
| ALT (IU/L)^a^ | 20.94 (12.31) | 26.08 (23.18) | 26.97 (16.89) | <0.001 | a,b |
| AST (IU/L)^a^ | 24.05 (8.73) | 26.81 (16.20) | 28.43 (16.59) | <0.001 | a,b |
| hsCRP (mg/L)^a^ | 1.63 (5.60) | 1.54 (2.15) | 2.53 (7.16) | <0.001 | a,b,c |
| Smoking status (%)  Non-smoker  Ex-smoker  Current smoker | 611 (66.5%)  158 (17.2%)  150 (16.3%) | 167 (68.4%)  38 (15.6%)  39 (16.0%) | 222 (59.5%)  92 (24.7%)  59 (15.8%) | 0.017 |  |
| Alcohol intake (%)  Non-drinker  Ex-drinker  Current drinker | 457 (49.7%)  45 (4.9%)  417 (45.4%) | 131 (53.7%)  16 (6.6%)  97 (39.7%) | 190 (50.9%)  35 (9.4%)  148 (38.7%) | 0.018 |  |
| Exercise (%) | 298 (32.4%) | 73 (29.9%) | 110 (29.5%) | 0.515 |  |

Subjects in diabetes (diabetes) were newly diagnosed and untreated. In *post hoc* analysis, each lower-case letter in the last column represents a pair with a significant difference; ‘a’ for NGT and IGT; ‘b’ for NGT and diabetes; ‘c’ for IGT and diabetes.

^a^Variable was log2-transformed before statistical analysis. The mean and SD of the log2-transformed variables were back-transformed to the original unit (for example, presented mean was back-transformed from $2^{mean of {log}_{2} FPG}$).

**p* values were calculated by one-way analysis of variance, with Tukey’s *post hoc* test used to determine differences among the groups.

FPG, fasting plasma glucose; BP, blood pressure; ALT, alanine aminotransferase; AST, aspartate aminotransferase; hsCRP, high-sensitivity C-reactive protein.

**Supplementary Table S2. Correlations of AhRL, MIS-ATP, and MIS-ROS with clinical parameters across the whole group according to gender**

|  | Total | | Male | | Female | |
| --- | --- | --- | --- | --- | --- | --- |
|  | r | *p* value | r | *p* value | r | *p* value |
| AhRL (pM, TCDDeq) |  |  |  |  |  |  |
| Age | 0.17 | <0.001 | 0.12 | 0.001 | 0.18 | <0.001 |
| BMI (kg/m^2^) | 0.15 | <0.001 | 0.16 | <0.001 | 0.15 | <0.001 |
| Weight (kg) | 0.09 | <0.001 | 0.12 | 0.001 | 0.06 | 0.102 |
| Waist circumference (cm) | 0.20 | <0.001 | 0.21 | <0.001 | 0.2 | <0.001 |
| Systolic BP (mm Hg) | 0.15 | <0.001 | 0.18 | <0.001 | 0.18 | <0.001 |
| Diastolic BP (mm Hg) | 0.05 | 0.048 | 0.03 | 0.384 | 0.07 | 0.058 |
| HbA_1c_ (%)* | 0.51 | <0.001 | 0.47 | <0.001 | 0.47 | <0.001 |
| FPG (mmol/L)* | 0.41 | <0.001 | 0.47 | <0.001 | 0.36 | <0.001 |
| 2h glucose (mmol/L)* | 0.51 | <0.001 | 0.47 | <0.001 | 0.5 | <0.001 |
| Fasting insulin (mmol/L)* | 0.15 | <0.001 | 0.17 | <0.001 | 0.15 | <0.001 |
| HOMA-IR* | 0.28 | <0.001 | 0.32 | <0.001 | 0.26 | <0.001 |
| HOMA-β* | –0.23 | <0.001 | -0.24 | <0.001 | -0.19 | <0.001 |
| Total cholesterol (mg/dL) | 0.03 | 0.245 | 0.02 | 0.608 | 0.04 | 0.268 |
| Triglyceride (mg/dL)* | 0.23 | <0.001 | 0.19 | <0.001 | 0.22 | <0.001 |
| HDL cholesterol (mg/dL)* | –0.13 | <0.001 | -0.11 | 0.003 | -0.13 | <0.001 |
| LDL cholesterol (mg/dL)* | –0.05 | 0.071 | -0.07 | 0.082 | -0.01 | 0.706 |
| ALT (IU/L)* | 0.19 | <0.001 | **0.06** | **0.144** | **0.21** | **<0.001** |
| AST (IU/L)* | 0.12 | <0.001 | **-0.02** | **0.545** | **0.14** | **<0.001** |
| hsCRP (mg/L)* | 0.13 | <0.001 | 0.1 | 0.007 | 0.15 | <0.001 |
|  |  |  |  |  |  |  |
| MIS-ATP (%) |  |  |  |  |  |  |
| Age | -0.13 | <0.001 | -0.09 | 0.015 | -0.16 | <0.001 |
| BMI (kg/m^2^) | -0.1 | <0.001 | -0.06 | 0.139 | -0.14 | <0.001 |
| Weight (kg) | -0.06 | 0.022 | -0.03 | 0.405 | -0.08 | 0.014 |
| Waist circumference (cm) | -0.15 | <0.001 | -0.11 | 0.006 | -0.19 | <0.001 |
| Systolic BP (mm Hg) | -0.14 | <0.001 | -0.08 | 0.036 | -0.18 | <0.001 |
| Diastolic BP (mm Hg) | -0.06 | 0.027 | 0 | 0.996 | -0.1 | 0.002 |
| HbA_1c_ (%)* | -0.29 | <0.001 | -0.26 | <0.001 | -0.31 | <0.001 |
| FPG (mmol/L)* | -0.28 | <0.001 | -0.26 | 0.001 | -0.29 | <0.001 |
| 2h glucose (mmol/L)* | -0.36 | <0.001 | -0.36 | <0.001 | -0.36 | <0.001 |
| Fasting insulin (mmol/L)* | -0.11 | <0.001 | -0.09 | 0.017 | -0.13 | <0.001 |
| HOMA-IR* | -0.2 | <0.001 | -0.18 | <0.001 | -0.22 | <0.001 |
| HOMA-β* | 0.15 | <0.001 | 0.15 | <0.001 | 0.15 | <0.001 |
| Total cholesterol (mg/dL) | 0.01 | 0.627 | -0.01 | 0.814 | 0.03 | 0.439 |
| Triglyceride (mg/dL)* | -0.18 | <0.001 | -0.18 | <0.001 | -0.17 | <0.001 |
| HDL cholesterol (mg/dL)* | 0.09 | <0.001 | 0.07 | 0.052 | 0.11 | 0.001 |
| LDL cholesterol (mg/dL)* | 0.07 | 0.003 | 0.09 | 0.021 | 0.06 | 0.073 |
| ALT (IU/L)* | -0.12 | <0.001 | **-0.05** | **0.236** | **-0.19** | **<0.001** |
| AST (IU/L)* | -0.1 | <0.001 | **-0.07** | **0.058** | **-0.13** | **<0.001** |
| hsCRP (mg/L)* | -0.06 | 0.013 | **-0.05** | **0.156** | **-0.07** | **0.043** |
| MIS-ROS (%) |  |  |  |  |  |  |
| Age | 0.04 | 0.139 | 0.01 | 0.807 | 0.06 | 0.07 |
| BMI (kg/m^2^) | 0.1 | <0.001 | 0.15 | <0.001 | 0.08 | 0.015 |
| Weight (kg) | 0.09 | <0.001 | 0.1 | 0.009 | 0.06 | 0.07 |
| Waist circumference (cm) | 0.15 | <0.001 | 0.18 | <0.001 | 0.12 | <0.001 |
| Systolic BP (mm Hg) | 0.11 | <0.001 | 0.09 | 0.017 | 0.13 | <0.001 |
| Diastolic BP (mm Hg) | <0.01 | 0.97 | -0.01 | 0.841 | 0 | 0.957 |
| HbA_1c_ (%)* | 0.23 | <0.001 | 0.24 | <0.001 | 0.23 | <0.001 |
| FPG (mmol/L)* | 0.31 | <0.001 | 0.34 | <0.001 | 0.28 | <0.001 |
| 2h glucose (mmol/L)* | 0.35 | <0.001 | 0.42 | <0.001 | 0.29 | <0.001 |
| Fasting insulin (mmol/L)* | 0.16 | <0.001 | 0.18 | <0.001 | 0.15 | <0.001 |
| HOMA-IR* | 0.26 | <0.001 | 0.28 | <0.001 | 0.24 | <0.001 |
| HOMA-β* | -0.13 | <0.001 | -0.13 | <0.001 | -0.12 | <0.001 |
| Total cholesterol (mg/dL) | -0.03 | 0.188 | 0.01 | 0.786 | -0.06 | 0.077 |
| Triglyceride (mg/dL)* | 0.13 | <0.001 | 0.18 | <0.001 | 0.08 | 0.013 |
| HDL cholesterol (mg/dL)* | -0.12 | <0.001 | -0.12 | 0.002 | -0.11 | <0.001 |
| LDL cholesterol (mg/dL)* | -0.07 | 0.003 | -0.07 | 0.073 | -0.07 | 0.044 |
| ALT (IU/L)* | 0.03 | 0.311 | -0.02 | 0.585 | 0.05 | 0.11 |
| AST (IU/L)* | -0.02 | 0.443 | -0.06 | 0.147 | 0 | 0.967 |
| hsCRP (mg/L)* | 0.11 | <0.001 | 0.14 | <0.001 | 0.09 | 0.011 |

Correlation coefficients (r) and *p* values were calculated using Pearson’s correlation analysis.

*Variable was log2-transformed before statistical analysis. G

FPG, fasting plasma glucose; BP, blood pressure; ALT, alanine aminotransferase; AST, aspartate aminotransferase; hsCRP, high-sensitivity C-reactive protein.

**Supplementary Table S3. Comparison of AhRL, MIS-ATP, and MIS-ROS according to sex, and smoking, alcohol intake, and exercise status**

|  |  | AhRL  (pM, TCDDeq) | | | MIS-ATP  (% of CSS) | | | MIS-ROS  (% of CSS) | | |
| --- | --- | --- | --- | --- | --- | --- | --- | --- | --- | --- |
|  | n | mean (±SD) | *p*  value | Post hoc | mean (±SD) | *p*  value | Post hoc | mean (±SD) | *p*  value | *Post hoc* |
| **Sex**  Male  Female | 687  850 | 2.78 (1.83)  2.62 (1.82) | 0.092 |  | 88.98 (12.92)  89.32 (12.58) | 0.608 |  | 116.60 (16.01)  115.40 (15.13) | 0.134 |  |
| **Smoking**  Never  Ex  Current | 1000  286  249 | 2.61 (1.79)  2.95 (1.96)  2.74 (1.79) | 0.019 | a | 89.35 (12.53)  89.16 (13.52)  88.49 (12.64) | 0.631 |  | 115.47 (15.35)  118.17 (15.87)  115.15 (15.73) | 0.024 | a, c |
| **Drinker**  Never  Ex  Current | 778  96  662 | 2.67 (1.82)  3.16 (1.98)  2.65 (1.80) | 0.032 | a, c | 89.30 (12.52)  87.30 (12.87)  89.30 (12.96) | 0.331 |  | 116.34 (15.91)  117.32 (15.18)  115.23 (15.12) | 0.268 |  |
| **Exercise**  No  Yes | 1055  481 | 2.75 (1.86)  2.56 (1.75) | 0.061 |  | 89.21 (12.41)  89.09 (13.42) | 0.869 |  | 114.92 (15.26)  118.13 (15.90) | <0.001 |  |

Significant differences: ‘a’ for never and ex; ‘b’ for never and current; ‘c’ for ex and current.

*p* values were calculated by one-way analysis of variance followed by Tukey’s *post hoc* test.

AhRL is expressed as 2,3,7,8-tetrachlorodibenzodioxin (TCDD) equivalents (TCDDeq, pM). MIS-ATP and MIS-ROS are expressed as % of the 10% charcoal-stripped human serum (CSS)-treated control.

**Supplementary Table S4. Relative risk (95% CI) of diabetes incidence within 4 years of non-diabetic subjects (n=1,163): combined effects of AhRL and MIS-ATP.**

|  | Group 1 (0)  (AhRL < 2.70,  MIS-ATP > 88.1) | Group 2 (10)  (AhRL < 2.70,  MIS-ATP ≤ 88.1) | Group 3 (1)  (AhRL ≥ 2.70,  MIS-ATP > 88.1) | Group 4 (11)  (AhRL ≥ 2.70,  MIS-ATP ≤ 88.1) |
| --- | --- | --- | --- | --- |
| Number |  |  |  |  |
| Total  Male  Female | 618  270  348 | 274  106  168 | 103  43  60 | 168  84  84 |
| Number of diabetes (%) |  |  |  |  |
| Total  Male  Female | 6 (1.0%)  4 (1.5%)  2 (0.6%) | 13 (4.7%)  5 (4.7%)  8 (4.8%) | 12 (11.7%)  6 (13.9%)  6 (10.0%) | 38 (22.6%)  25 (29.8%)  13 (15.5%) |
| Model A |  |  |  |  |
| Total  Male  Female | 1.0(ref)  1.0(ref)  1.0(ref) | 5.08 (1.91–13.51)  3.29 (0.87-12.50)  8.65 (1.82-41.20) | 13.45 (4.93–36.72)  10.78 (2.90-40.01)  19.22 (3.78-97.70) | 29.82 (12.35–71.99)  28.17 (9.45 –84.02)  31.68 (6.99-143.45) |
| Model B |  |  |  |  |
| Total  Male  Female | 1.0(ref)  1.0(ref)  1.0(ref) | 4.82 (1.80–12.91)  3.11 (0.82-11.86)  7.32 (1.51-35.59) | 13.72 (4.96–37.89)  11.49 (3.05-43.22)  15.62 (2.99-81.71) | 29.34 (12.05–71.44)  29.36 (9.76-88.35)  28.33 (6.17-129.95) |
| Model C |  |  |  |  |
| Total  Male  Female | 1.0(ref)  1.0(ref)  1.0(ref) | 4.84 (1.80–13.01)  3.23 (0.84-12.47)  7.67 (1.58-37.37) | 12.26 (4.38–34.31)  11.19 (2.86-43.87)  14.43 (2.75-75.72) | 26.12 (10.63–64.17)  30.35 (9.80-94.02)  23.99 (5.13-112.24) |
| Model D |  |  |  |  |
| Total  Male  Female | 1.0(ref)  1.0(ref)  1.0(ref) | 4.40 (1.61–11.99)  3.54 (0.89-14.08)  6.09 (1.22-30.50) | 9.17 (3.19–26.37)  9.64 (2.33-39.89)  10.36 (1.90-56.50) | 21.11 (8.46–52.67)  28.43 (8.83-91.58)  16.50 (3.42-79.67) |

Model A: unadjusted.

Model B: sex, age, and smoking-adjusted.

Model C: model B + waist circumference and systolic BP-adjusted.

Model D: model C + fasting glucose and triglyceride-adjusted.

Values are expressed as relative risk (95% confidence interval).

AhRL is expressed as 2,3,7,8-tetrachlorodibenzodioxin (TCDD) equivalents (TCDDeq, pM). MIS-ATP is expressed as % of the 10% charcoal-stripped human serum (CSS)-treated control.

**Supplementary Methods**

***Study participants***

The Ansung cohort of KoGES was established to investigate the genetic and environmental aetiology of common, complex diseases in Koreans[^1^](#_ENREF_1)^,^[^2^](#_ENREF_2). Ansung is a representative rural farming community and had a population of 132,906 in 2000. Using cluster sampling according to age, sex, and residential district in Ansung, 5,018 of 7,192 eligible subjects were investigated during the baseline year 2002 (response rate = 69.6%), then follow-up examinations were conducted in 2-year intervals. The eligibility criteria at baseline included an age of 40–69 years, residence within the borders of the survey area for at least 6 months before testing, and sufficient mental and physical ability to participate.

Ansung study focused on diabetes and its related problems, such that every participant underwent the 75g oral glucose tolerance test (OGTT), if they had neither been diagnosed with diabetes nor were on antidiabetic medication. We analysed 1,537 sera collected during OGT testing in 2008. Of the corresponding participants, 919 had normal glucose tolerance (NGT), 244 had IGT, and 374 had diabetes. The definitions of NGT, IGT, and diabetes were based on the results of the 75g OGTT and the WHO criteria[^3^](#_ENREF_3)^,^[^4^](#_ENREF_4). In brief, NGT was defined as <126 mg/dL in fasting plasma glucose (FPG) and <140 mg/dL in postload 2-h glucose concentrations after OGTT (2h glucose). IGT was defined as FPG<126 mg/dL and 200 mg/dL> 2h glucose ≥140 mg/dL; diabetes was defined as FPG ≥126 mg/dL, or 2h glucose ≥200 mg/dL, or on antidiabetic medication.

The results of KoGES are open to the public, and a summary of this study was recently published[^1^](#_ENREF_1). The data used in this study were downloaded from the KoGES depository at the National Research Institute of Health, Centres for Disease Control and Prevention, Ministry for Health and Welfare, Republic of Korea, with permission (KoGES; 4851-302). The study protocol was approved by the Ethics committee of the Institutional Review Board of Ajou University School of Medicine (IRB No. AJIRB-CRO-07-012), Kyung Hee University (IRB No. KHSIRB-14-056), and Eulji University College of Medicine (IRB No. EGH2015-12-005).

***Clinical characteristics and parameters***

KoGES investigators measured anthropometric parameters and blood pressure by standard methods at local health research centres. Blood samples were transported to a central laboratory, where all clinical parameters were measured or calculated. These data were then compared with data obtained from the follow-up study conducted during 2012. The fasting plasma concentrations of glucose, creatinine, alanine and aspartate aminotransferases (ALT and AST, respectively), total cholesterol, triglycerides (TG), and high-density lipoprotein (HDL)-cholesterol were measured using a Hitachi 747 chemistry analyser (Hitachi Ltd, Tokyo, Japan). The low-density lipoprotein (LDL)–cholesterol level (mg/dL) was derived using the following formula: total cholesterol [mg/dL] − HDL-cholesterol [mg/dL] − triglyceride [mg/dL]/5. The glycosylated haemoglobin (HbA1c) level was determined by high-performance liquid chromatography (Variant II; BioRad Laboratories, Hercules, CA, USA). High-sensitivity C-reactive protein (hsCRP) was measured by immunoradiometric assay (ADVIA 1650, Bayer Diagnostics, Tarrytown, NY, USA) and plasma insulin concentration by radioimmunoassay (Linco kit, St Charles, MO, USA).

To evaluate insulin resistance. the homeostasis model assessment of β-cell function (HOMA-β) was calculated as 20×(fasting insulin [μU/mL])/(fasting glucose – 3.5 [mmol/L]), and the homeostasis model assessment of insulin resistance index (HOMA-IR) was calculated as (fasting glucose [mmol/L]) × (fasting insulin [μU/mL])/22.5[^2^](#_ENREF_2)^,^[^5^](#_ENREF_5). The insulinogenic index (IGI), an estimate of early insulin secretion, was calculated by dividing the increase in insulin during the first 60 min by the increase in glucose during the same period [60 min − 0 min insulin (IU/mL)]/[60 min − 0 min glucose (mg/mL)][^6^](#_ENREF_6). The composite insulin sensitivity index (ISI) of Matsuda, which includes measures of insulin sensitivity, was calculated following the original description[^7^](#_ENREF_7).

***AhR ligand (AhRL) bioactivity assay***

The pGL4-DRE-luc(puromycin+)/pRL-mTK double-positive stable cells and heat-inactivated serum samples were prepared as described previously[^8^](#_ENREF_8). The AhRL assay is similar to the CALUX assay, except adapting the different recombinant cell lines and organic solvent extraction-free sample preparation method[^9^](#_ENREF_9). All cell-based assays were performed in duplicate on blinded samples. The pGL4-DRE-luc/pRL-mTK-transfected mouse Hepa1c1c7 cells (1 × 10^5^/well) in a 96-well plate were treated for 24 h with 10 μL serum samples (10% of culture media) or control charcoal-stripped human serum (CSS) in phenol red-free Dulbecco’s modified Eagle’s medium. Luciferase activity was measured using a Dual-Glo Luciferase assay system (Promega, Madison, WI, USA) and a luminometer (Berthold, Bad Wildbad, Germany) and subsequently normalized against *Renilla* luciferase activity. The relative AhR-dependent luciferase reporter activity reflects the AhR ligand (AhRL) bioactivity of serum sample-treated cells, which was calculated as fold induction (FI), with the AhRL of the 10% CSS-treated cells being set as 1. Then, AhRL values were converted to 2,3,7,8-tetrachlorodibenzodioxin (TCDD) equivalents (TCDDeq, pM) using a standard curve (0–10 pM of TCDD), prepared using the AhRL of cells exposed to serially diluted TCDD (0–50 pM) for 24 hours in the presence of 10% CSS. The intra- and interassay coefficients of variation for these methods were less than 5.0%.

***MIS-ATP and MIS-ROS assays***

Levels of mitochondria-inhibiting substances (MIS) in serum samples were evaluated by measuring intracellular ATP content[^8^](#_ENREF_8) (MIS-ATP) and ROS generation (MIS-ROS) as biomarkers for mitochondrial function of cultured cells incubated with serum samples. Similarly to the AhRL assay, pRL-mTK-transfected mouse Hepa1c1c7 cells (5 × 10^4^/well) in a 96-well plate were treated with 10 μL heat-inactivated-serum samples for 48 h. The ATP content of the treated cells was determined using the luciferin-luciferase reaction with the CellTiter-Glo luciferase kit (Promega, Madison, WI, USA), with the output being normalized to *Renilla* luciferase activity. The intracellular ATP content of CSS-treated control cells was 65.1 ± 2.7 nM. The ATP concentration of 10% sample serum-treated cells can be calculated from the standard curve of ATP concentration (nM) = (% control + 18.24)/1.817. ROS generation was determined using 5-(and-6)-chloromethyl-2',7'-dichlorodihydrofluorescein diacetate and acetyl ester (CM-H_2_DCFDA; Molecular Probes, Eugene, OR, USA). The serum-treated cells were incubated with 1 μM CM-H_2_DCFDA and Hoechst 33342 (0.5 μM) for 1 h at 37°C. The fluorescence intensities at 485 nm/535 nm for DCF-DA were normalized by the Hoechst intensity at 355 nm/460 nm.

Both MIS-ATP and MIS-ROS were expressed as % of CSS-treated control. The intra- and interassay coefficients of variation for these methods were less than 6.0%.

***Statistical analysis***

The data were analysed using SPSS23 software. All variables were evaluated for normality using the Shapiro-Wilk test, and variables with non-Gaussian distribution (fasting insulin, triglycerides, HOMA-β, HOMA-IR, composite insulin sensitivity index, HDL, HbA_1c_, fasting plasma glucose, 2 h glucose, ALT, AST, and hsCRP) were log2-transformed. Results are presented as means ± standard deviation (SD), as numbers and percentages, or as a relative risk (RR) with 95% confidence intervals (CIs). Students’ *t*-tests or analysis of variance were used to compare the means of continuous variables among the groups. Tukey *post hoc* tests were performed to assess which pairs of groups showed significant differences. Comparisons of the proportions of categorical variables among the groups were performed using the χ^2^ test or logistic regression. Optimal cut-off values of AhRL and MIS-ATP for the prediction of diabetes development were estimated using receiver-operating characteristics (ROCs) and the Youden method[^10^](#_ENREF_10). The relative risks (RRs) of participants with AhRL or MIS-ATP above the ROC cutoff values for diabetes development within 4 years were analysed with logistic regression, with or without adjustment for potentially confounding variables: model A, unadjusted; model B, adjusted for sex, age, family history of diabetes, smoking, drinking, and exercise; model C, model B adjusted for waist circumference and systolic blood pressure; model D, model C adjusted for HbA_1c_, HOMA-IR, HOMA-β-cell function, total cholesterol, triglycerides, HDL, ALT, and hsCRP. Statistical significance was defined as a two-tailed p **<** 0.05.

The measured AhRL, MIS-ATP, and MIS-ROS were clustered using the K-means clustering algorithm[^11^](#_ENREF_11). The K-means algorithm finds *k* clusters by iterative updates of the cluster centres in order to minimize the variances within each cluster. Here, *k* = 2 was used. For the clustering, each variable was log-transformed and standardized with a zero mean and unit variance.

**References**

1 Kim, Y., Han, B. G. & Ko, G. E. S. g. Cohort Profile: The Korean Genome and Epidemiology Study (KoGES) Consortium. *International journal of epidemiology* **46**, e20, doi:10.1093/ije/dyv316 (2017).

2 Ohn, J. H. *et al.* 10-year trajectory of beta-cell function and insulin sensitivity in the development of type 2 diabetes: a community-based prospective cohort study. *Lancet Diabetes Endocrinol* **4**, 27-34, doi:10.1016/S2213-8587(15)00336-8 (2016).

3 Alberti, K. G. & Zimmet, P. Z. Definition, diagnosis and classification of diabetes mellitus and its complications. Part 1: diagnosis and classification of diabetes mellitus provisional report of a WHO consultation. *Diabetic medicine : a journal of the British Diabetic Association* **15**, 539-553, doi:10.1002/(SICI)1096-9136(199807)15:7<539::AID-DIA668>3.0.CO;2-S (1998).

4 The Expert Committee on the, of, D. a. C. & Mellitus, D. Report of the expert committee on the diagnosis and classification of diabetes mellitus. *Diabetes Care* **26 Suppl 1**, S5-20 (2003).

5 Matthews, D. R. *et al.* Homeostasis model assessment: insulin resistance and beta-cell function from fasting plasma glucose and insulin concentrations in man. *Diabetologia* **28**, 412-419 (1985).

6 Tura, A., Kautzky-Willer, A. & Pacini, G. Insulinogenic indices from insulin and C-peptide: comparison of beta-cell function from OGTT and IVGTT. *Diabetes Res Clin Pract* **72**, 298-301, doi:10.1016/j.diabres.2005.10.005 (2006).

7 Matsuda, M. & DeFronzo, R. A. Insulin sensitivity indices obtained from oral glucose tolerance testing - Comparison with the euglycemic insulin clamp. *Diabetes Care* **22**, 1462-1470, doi:DOI 10.2337/diacare.22.9.1462 (1999).

8 Park, W. H. *et al.* Relationships between serum-induced AhR bioactivity or mitochondrial inhibition and circulating polychlorinated biphenyls (PCBs). *Sci Rep* **7**, 9383, doi:10.1038/s41598-017-09774-1 (2017).

9 Vondracek, J. *et al.* Assessment of the aryl hydrocarbon receptor-mediated activities of polycyclic aromatic hydrocarbons in a human cell-based reporter gene assay. *Environ Pollut* **220**, 307-316, doi:10.1016/j.envpol.2016.09.064 (2017).

10 Ruopp, M. D., Perkins, N. J., Whitcomb, B. W. & Schisterman, E. F. Youden Index and optimal cut-point estimated from observations affected by a lower limit of detection. *Biometrical journal. Biometrische Zeitschrift* **50**, 419-430, doi:10.1002/bimj.200710415 (2008).

11 Cheng, J. *et al.* Tryptophan derivatives regulate the transcription of Oct4 in stem-like cancer cells. *Nature communications* **6**, 7209, doi:10.1038/ncomms8209 (2015).
